# More Than Spikes: On the Added Value of Non-linear Intracranial EEG Analysis for Surgery Planning in Temporal Lobe Epilepsy

**DOI:** 10.3389/fneur.2021.741450

**Published:** 2022-01-13

**Authors:** Michael Müller, Martijn Dekkers, Roland Wiest, Kaspar Schindler, Christian Rummel

**Affiliations:** ^1^Support Center for Advanced Neuroimaging (SCAN), University Institute for Diagnostic and Interventional Neuroradiology, Inselspital, Bern, Switzerland; ^2^Department of Neurology, Inselspital, Bern University Hospital, University Bern, Bern, Switzerland

**Keywords:** epilepsy, epileptiform events, quantitative EEG, epilepsy surgery, non-linear interrelations

## Abstract

Epilepsy surgery can be a very effective therapy in medication refractory patients. During patient evaluation intracranial EEG is analyzed by clinical experts to identify the brain tissue generating epileptiform events. Quantitative EEG analysis increasingly complements this approach in research settings, but not yet in clinical routine. We investigate the correspondence between epileptiform events and a specific quantitative EEG marker. We analyzed 99 preictal epochs of multichannel intracranial EEG of 40 patients with mixed etiologies. Time and channel of occurrence of epileptiform events (spikes, slow waves, sharp waves, fast oscillations) were annotated by a human expert and non-linear excess interrelations were calculated as a quantitative EEG marker. We assessed whether the visually identified preictal events predicted channels that belonged to the seizure onset zone, that were later resected or that showed strong non-linear interrelations. We also investigated whether the seizure onset zone or the resection were predicted by channels with strong non-linear interrelations. In patients with temporal lobe epilepsy (32 of 40), epileptic spikes and the seizure onset zone predicted the resected brain tissue much better in patients with favorable seizure control after surgery than in unfavorable outcomes. Beyond that, our analysis did not reveal any significant associations with epileptiform EEG events. Specifically, none of the epileptiform event types did predict non-linear interrelations. In contrast, channels with strong non-linear excess EEG interrelations predicted the resected channels better in patients with temporal lobe epilepsy and favorable outcome. Also in the small number of patients with seizure onset in the frontal and parietal lobes, no association between epileptiform events and channels with strong non-linear excess EEG interrelations was detectable. In contrast to patients with temporal seizure onset, EEG channels with strong non-linear excess interrelations did neither predict the seizure onset zone nor the resection of these patients or allow separation between patients with favorable and unfavorable seizure control. Our study indicates that non-linear excess EEG interrelations are not strictly associated with epileptiform events, which are one key concept of current clinical EEG assessment. Rather, they may provide information relevant for surgery planning in temporal lobe epilepsy. Our study suggests to incorporate quantitative EEG analysis in the workup of clinical cases. We make the EEG epochs and expert annotations publicly available in anonymized form to foster similar analyses for other quantitative EEG methods.

## 1. Introduction

Surgical removal of seizure-generating brain tissue is an established and often beneficial treatment option for patients with drug-resistant epilepsy. Identification of the tissue necessary and sufficient to cease seizure activity [the “epileptogenic zone,” EZ, (1, 2)] is essential but challenging, especially if there is no obvious anatomical correlate (as e.g., a brain lesion or tumor).

The decision on which area to resect and how this will putatively influence epileptic activity is individually determined for each patient, taking various diagnostic information sources into consideration (including scalp EEG, structural and functional MRI, psychological assessments and intracranial EEG if necessary). Although these assessments usually follow established concepts like the importance of the “seizure onset zone” (SOZ), which is used as a proxy for the EZ, the procedure suffers from a considerable amount of subjectivity. The limitations of the current approaches regarding reliable prediction of the patients' benefit from epilepsy surgery are apparent, since only about half of all patients undergoing surgery become permanently seizure free, a rate that has practically not improved over decades (3–10). This issue is strongly associated with the heterogeneity of the disorder. It is now generally accepted that epilepsy needs to be considered a network-based disease, characterized by the interaction of multiple brain regions (11–15). Due to the lack of methodologies allowing to record brain activity with simultaneously high spatial and temporal resolution and full coverage, seizure dynamics are still not understood in full detail.

Intracranial EEG (iEEG) is currently the gold standard in this regard, providing excellent temporal and spatial resolution with the drawback of limited spatial coverage. However, purely visual analysis is limited due to the abundance of potential interrelations between dozens of iEEG channels (16), exacerbated by the ever-growing amount of data due to increasing spatial resolution (17) and recording time (18, 19). Quantitative EEG (qEEG) methods, capable of capturing a variety of signal properties (including very complex, visually undetectable ones) and processing large amounts of data have been presented over the last decades. They hold great promise to discern and provide additional information and ultimately increase the success rate of epilepsy surgery. High frequency oscillations (80–500 Hz) have long been considered a promising marker of the EZ (20–22). Along with the network conception of epilepsy, multivariate methods, quantifying signal dependencies, gained increasing attention [see (23, 24) for reviews]. In congruence with the strong evidence for high non-linearity of epileptic activity (25–29), segregated non-linear signal dependencies have been demonstrated to contain relevant information (27, 30).

Despite a large variety of qEEG measures have shown to capture some disease-related properties, none is applied in clinical routine to date. Besides undefined standardization of methods and interpretation and a lack of implementation in certified software, one reason might be the suspicion that qEEG markers could only be sensitive to signal features that are similarly detectable by visual expert analysis, like e.g., frequent interictal spikes (31). Thus, besides an extensive evaluation of qEEG measures, a better understanding to what extent these are related to traditional markers of epilepsy and what they might reflect beyond, will be very helpful in the effort to gain clinical acceptance.

Strictly speaking, the epileptogenicity of brain tissue (i.e., its belonging to the EZ, a theoretical concept) is not accessible by iEEG or any other current mapping technique. This implies that when aiming to identify markers that are closely associated with the brain tissue's epileptogenicity, one is confronted with the problem of a missing ground truth. The SOZ can be visually determined by experts from the transition from preictal to ictal EEG *before* surgery (i.e., agnostic of post-surgical seizure control) but is known to be only an approximation of the EZ (1). In addition, the extent of its observation depends on electrode placement. On the other hand, the resected brain tissue (RBT) is available only in patients who undergo surgery, often larger than minimally required and (by definition) fully contains the EZ only in patients who became seizure free. From this we hypothesized that the ability of any marker of epileptogenicity to determine the RBT should be larger in patients who became seizure free after surgery than in patients with unfavorable outcome. In contrast, the agreement between the SOZ and any marker of epileptogenicity might depend on post-surgical seizure control only indirectly.

In our previous study (32), we have demonstrated for patients with mesiotemporal implantation of depth electrodes that surrogate corrected non-linear interrelations between iEEG signals were associated with the individual pathology. In addition, the spatial overlap of salient non-linear interrelations with the RBT was associated with post-surgical seizure control. Here, we investigated the relationship between the occurrence of salient non-linear interrelations and traditional markers of epileptogenicity (33), namely spikes, slow waves, sharp waves, and fast oscillations, as identified by a human expert. In addition, we determined the relationship with the SOZ and where applicable with the RBT. We manually annotated and analyzed 99 preictal epochs of multi-channel iEEG from 40 patients including several types of epilepsy syndromes and etiologies as well as electrode implantation schemes beyond mesio-temporal depth electrodes. To reduce data heterogeneity, we limited our main analyses to patients with seizure onset in the temporal lobe, which were the vast majority in our dataset (32/40). Explorative examination of patients with a non-temporal seizure onset (six frontal, two parietal) are provided in the Supplementary Materials. To assess the association of epileptogenic brain tissue with EEG markers (be they visual or quantitative), we contrasted accuracy quantifiers on the channel level between patients with favorable and unfavorable post-surgical outcomes.

To foster similar analyses, we make the full EEG recordings and expert annotations used in this paper publicly available in anonymized form via GitHub together with a custom EEG reader (github.com/SCAN-NRAD/scanEEGviewer).

## 2. Methods

### 2.1. Patients and Data

We included data of 40 patients with drug-resistant epilepsies, who were considered for epilepsy surgery at the Inselspital Bern (58% female, median age 35 years, IQR 19 years, range 9−66 years). More detailed patient information is provided in Table 1. To characterize success of the intervention, we used the first available outcome assessment at least three months after surgery according to the Engel scale. The first assessment is most representative of the direct effects of the surgery, not influenced by subsequent effects like neuronal plasticity and changes in patient compliance, which might change the long-term outcome but are hardly predictable. In total, 19 patients became completely seizure free (Engel class I), 4 patients became almost seizure free (Engel class II), 4 patients had worthwhile improvement (Engel class III), and 7 patients had no improvement (Engel class IV). In the group analyses presented in the main text we only included patients with seizure onset in the temporal lobe to preserve data homogeneity. The results for the remaining patients are compiled in the Supplementary Materials. At the same time, to increase sample size, we dichotomized outcomes into “favorable” (Engel classes I & II) and “unfavorable” (Engel classes III & IV). Using coregistration of a post-implantation CT and a postsurgical MRI, the patient-specific RBT and thereby the iEEG channels recording from this tissue were determined [see (34) for a detailed description of this procedure].

**Table 1:** Patients included in this study.

	**Engel**	**epoch**	**Seizure**		**Type of**	**# of**	**# of**
**Patient**	**class**	**dur. [s]**	**onset**	**Histology/MRI**	**resection**	**channels**	**ch. in RBT**
p1	I	180/176/182	T (B)	Non-lesional	TPE + SAHE (R)	102	13
*p2	I	182/177	T (R)	Hippocampal sclerosis	T2/3E	32	5
*p3	I	199/179	T (R)	Hippocampal sclerosis	T2/3E	38	8
*p4	I	179/170	T (R)	Hippocampal sclerosis	SAHE	31	13
*p5.1	I	167/169	F (L)	Ectopic Neurons	LE SMA	76	7
p5.2	I	177/176	F (L)	Ectopic Neurons	LE SMA	86	14
*p6	I	170/180	T (R)	Hippocampal sclerosis	SAHE	32	7
p7	I	180/177/181	T (L)	Hippocampal sclerosis	LE	37/35/34	8/8/7
p8	I	185/183	T (L)	Hippocampal sclerosis	T2/3	64	13
p9	I	197/188	T (L)	Non-lesional	TLE	56	5
p10	I	176/184/180	T (R)	Hippocampal sclerosis	SAHE	34	10
*p11	I	140/129/123	T (R)	Hippocampal sclerosis	TPE + SAHE	37/38/38	9
p12	I	178/166	T (L)	Glioma	LE T	74	13
p13	I	147/155	F (R)	Hemorrhage	LE	80	6
p14	I	115/122	T (L)	Bilateral HC sclerosis	SAHE	59	17
p15	I	185/171/179	T (L)	Hippocampal sclerosis	LE T	40	11
*p16	I	164/185	T (R)	Hippocampal sclerosis	LE MT	32	4
p17	I	180/181	F (R)	Non-lesional, mild FCD	LE	66	5
*p18	I	151/166	T (L)	Hippocampal sclerosis	SAHE	31	7
*p19	I	174/177	F (L)	Post-traumatic lesion	LE F	88	7
*p20	II	177/182	T (L)	Hippocampal sclerosis	TLE + SAHE	48	7
p21	II	186/180	T (L)	Other abnormal	TLE + SAHE	32	16
p22	II	183/180	T (R)	Non-lesional	SAHE	99	11
*p23	II	185/182/187	T (L)	Post-ischemic cyst	LE MT	29	2
*p24	III	180/179/158	F (L)	Non-lesional, FCD Ib	LE	69/70/70	6/4/4
p25	III	188/180	F (R)	FCD II	LE F+T	92	8
*p26	III	154/186	T (L)	Non-lesional	T2/3E	32	9
*p27	III	158/186/182	T (R)	Discrete alterations	SAHE	76	16
p28.1	IV	180/179	T (L)	Other abnormal	LE	59	2
p29.1	IV	182/180/183	T (L)	Non-lesional, Meningitis	TLE	61	10
p29.2	IV	168/168/165	T (L)	Non-lesional, Meningitis	TLE	48	8
p30	IV	179/179	T (R)	Non-lesional, Gliosis	T2/3E	100	13
p31	IV	113/112	T (L)	MT asymmetry	T2/3E	49	8
p32	IV	181/178/180	P (L)	MT asymmetry	LE	92/94/94	4
p33	IV	113/120	T (L)	FCD IIb	LE MT	24	6
*p34	IV	182/184	T (B)	MT sclerosis	SAHE (R)	32	14
*p28.2		179/178	T (L)	Other abnormal		64	
*p35		180/180/177	T (B)	Thickened MT structures		32	
p36		180/189	T (L)	TO Pachygyria right		59	
p37		129/177	P (L)	FCD		68	
*p38		197/180/195	T (R)	Non-lesional		24	
*p39		178/181	T (B)	Other abnormal		32	
p40		179/180	T (R)	MT asymmetry		32	

Indicated are the post-surgical seizure control according to the Engel classification scheme, durations of the included epochs, the location of seizure onset, etiological factors, the type of resection, the total number of artifact free channels, and the number of channels recording from RBT. Nineteen of these patients were already included in our preceding study (32) and are indicated by an asterisk *. One patient (p5) had two implantation schemes before resection, one patient (p29) had two distinct implantation schemes both followed by resection, and one patient (p28) had a second implantation after the surgical removal of brain tissue but no second resection.

*L, left; R, right; B, bilateral; T, temporal; F, frontal; P, parietal; FCD, focal cortial dysplasia; SMA, supplementary motor area; LE, lesionectomy; TLE, temporal lobectomy; TPE, temporal pole-ectomy; T2/3E, temporal 2/3 resection; SAHE, selective amygdala-hippocampectomy*.

This study was approved by the Ethics Committee of the Kanton of Bern (approval number 2017-00697). All decisions regarding the actual treatment of the patients (especially implantation and resection) were made solely on clinical grounds prior to this retrospective study and all patients gave written and informed consent that EEG and imaging data may be used for research purposes.

### 2.2. EEG Data, Epoch Selection, and Manual Annotation

EEG data was recorded using a NicoletOneTM recording system with a C64 amplifier (VIASYS Healthcore Inc., Madison, Wisconsin, USA) and intracranial depth, strip, and grid electrodes (AD-TECH, Wisconsin, USA). The sampling rate was 512 or 1,024 Hz, depending on whether more or less than 64 channels were used. Signals were referenced to an extracranial electrode (localized between 10–20 positions Fz and Cz) during recording and later re-referenced against the median of all artifact free channels. In addition, signals were band-pass filtered between 0.5 and 150 Hz using a fourth-order Butterworth filter.

Since the extensive manual annotation of iEEG data, typically comprising between 50 and 60 channels, is very time-consuming, it was impossible to analyze the entire long-term EEG recordings. In addition, the calculation of the qEEG measure used in this study (see below) is computationally expensive and prohibits long-term analysis beyond the range of minutes. In consequence, a compromise between epoch duration included per patient and the number of patients was inevitable. To minimize bias toward patients with many seizures we restricted the number of iEEG epochs per patient to at most three. All epochs had a duration between 110 and 200 s and ended at seizure onset. Permanently artifact corrupted channels (according to visual analysis by experts) were excluded from detailed visual or quantitative analysis (<5% of channels).

Several studies have shown that epilepsy dynamics underlie oscillations on various timescales, from circadian to multidien rhytms (35–39). Correspondingly, network measures calculated from iEEG data exhibit large circadian variations (40). To confine the arbitrariness of temporal data selection, we chose in each patient segments directly preceding the earliest artifact-free seizures recorded after implantation of the intracranial electrodes. This period serves as a relevant baseline for visual EEG analysis in clinical routine, and in contrast to ictal data, avoids artifacts that might be caused by seizure manifestation.

A clinical expert (M.D.) visually inspected all included iEEG epochs and manually annotated the extent of all epileptiform events (33) regarding time of occurrence and affected channels, corresponding to at least one of the following types: (1) spikes, (2) slow waves, (3) sharp waves, (4) fast oscillations. Channels were scored in a custom EEG-reader in referential mode. We scored pre-ictal transients as typical for epilepsy based on its sharp configuration and compared to the background activity in the same channel, looking either for high amplitudes or a disruption of ongoing rhythms. We distinguished spikes (duration <70 ms) and sharp waves (duration 70−200 ms). Slow wave activity was scored based on either a marked focal slowing of background activity, or the presence of slow waves with a high amplitude compared to the background activity. Fast oscillations were identified as episodes of focal activity with a frequency above 30 Hz. In addition, the channels comprising the seizure-specific SOZ were identified based on the presence of low-amplitude fast activity at seizure onset.

### 2.3. Non-linear Signal Dependence and Identification of Core Channels

The qEEG measure used in this study was introduced by Rummel et al. (34, 41) and has been applied in (32, 42, 43). A similar measure was also used in (16, 27, 30). In brief, non-linear interrelation matrices were determined by calculating mutual information of signal pairs over segments of 8 s duration, which were shifted over the entire epoch by 1-s steps. Mutual information quantifies the amount of information one signal provides about the other. Since it is sensitive to both linear an non-linear dependences alike, we used multivariate iterative amplitude adjusted Fourier transform (IAAFT) surrogate time series with conserved Pearson correlation matrix (44) to account for linear interrelation effects. Non-zero elements of the resulting interaction matrices had significantly stronger mutual information than the surrogate time series with conserved Pearson correlation. Hence, the matrices describe the non-linear *excess* interrelations, i.e., the interrelation that is not measurable by linear measures. To condense information, we averaged the resulting matrices over time. Since patient-wise contrasting of separate averages over segments with and without epileptiform events (45) were not consistently possible due to too dense or too sparse event occurrence in some patients, we averaged the interrelation matrices over the entire preictal epochs. From the resulting mean interrelation matrix, we calculated the normalized “node strength” (i.e., the mean interrelation of a channel with the remainder). This single value per channel is confined to the range [0, 1] and indicates how strongly it is connected with all others. Based on the channels' connection strength, we automatically separated the most strongly connected channels by sorting all channels by their node strengths and identifying the largest difference between two adjacent values on the linear and the logarithmic scale (32, 46). We call these epoch-specific channel collections the “core” and based on our previous findings (32, 34) hypothesized them to be indicative of pathological epileptic activity.

### 2.4. Statistical Analyses

To rule out the possibility of systematic differences, we compared the following quantifiers between patients of different outcome groups: total number of channels implanted, total number and relative portion of channels containing events, epoch-wise average number of channels per event, average event duration, total epoch duration. Likewise, we compared the total number and relative portion of resected channels, core channels, and channels constituting the SOZ and RBT. Moreover, we tested for different proportions of event types depending on the patients' post-surgical seizure control.

We used non-parametric testing throughout this study because sample sizes were small and distributions potentially skewed. Since patients who did not undergo surgery are likely a mixture of the favorable and unfavorable outcome groups with respect to surgery independent quantifiers, we excluded them from all outcome-dependent statistical comparisons, which enabled Mann-Whitney *U*-tests (MWU) between only two groups. Nevertheless, we display these data in our figures to document that this patient group did not behave systematically different. To compare event proportions between different groups we used Chi-squared tests.

The main objective of this study was to investigate the association between the channel-wise occurence of epileptiform events identified by expert EEG reading and sets of iEEG channels defined by the SOZ, the RBT, and the core channels of non-linear excess interrelation (see Figure S1 of the Supplementary Materials for an illustration), which either require information aggregation, surgical intervention or quantitative analysis. This was done by studying the degree to which one of these channel sets predicted another. Besides, we also examined dependences between these sets. Specifically, we defined a predictive set of iEEG channels, and a target set of channels. We then labeled all channels according to whether they were part of both sets (true positives, TP), only part of the predictive set (false positives, FP), only part of the target set (false negatives, FN), or not part of any set (true negatives, TN). Whenever epileptiform events were used as predictors, we performed this analysis separately for the four event types as well as for all types in aggregation. For every epoch and patient, we then pooled true/false positives/negatives over all events (same type and aggregated). Among the other channel sets, we determined for each epoch the predictive power of the presurgically defined SOZ for the RBT, which becomes available only after surgery. Similarly, as a specific example of qEEG analysis, we have assessed the predictive power of the core of non-linear excess interrelations for both the SOZ and the RBT.

Since events and the aforementioned sets of channels typically only comprised a minority of all iEEG channels, the number of TN by far exceeded those of the other categories in virtually all cases, heavily biasing all dependent accuracy measures. To avoid such bias, we report our results in terms of the TN-independent quantities precision and recall. As an overall accuracy quantifier we used their harmonic mean, the F1-score (see Supplementary Materials for details). All these quantifiers range in the interval [0, 1]. The precision (also called positive predictive value) specifies how indicative the predictive set is for the target set. Low values indicate that channels in the predictive set are often not part of the target set (many FP). Recall (also called sensitivity) specifies to what degree the target set is determined by the predictive set. Low values indicate that channels of the target set are often missed by the predictive set (many FN).

For all statistics we used an uncorrected significance level α = 0.01. Values *p* < 0.05 were interpreted as trends. Since each accuracy quantifier (precision, recall, F1-score) was tested for six different combinations of predictive and target sets, we applied Bonferroni correction when comparing predictions. The significance level was adjusted to α_Bonf_ = 0.0017 and values *p* < 0.0083 were interpreted as trends.

## 3. Results

**Figure 1:**
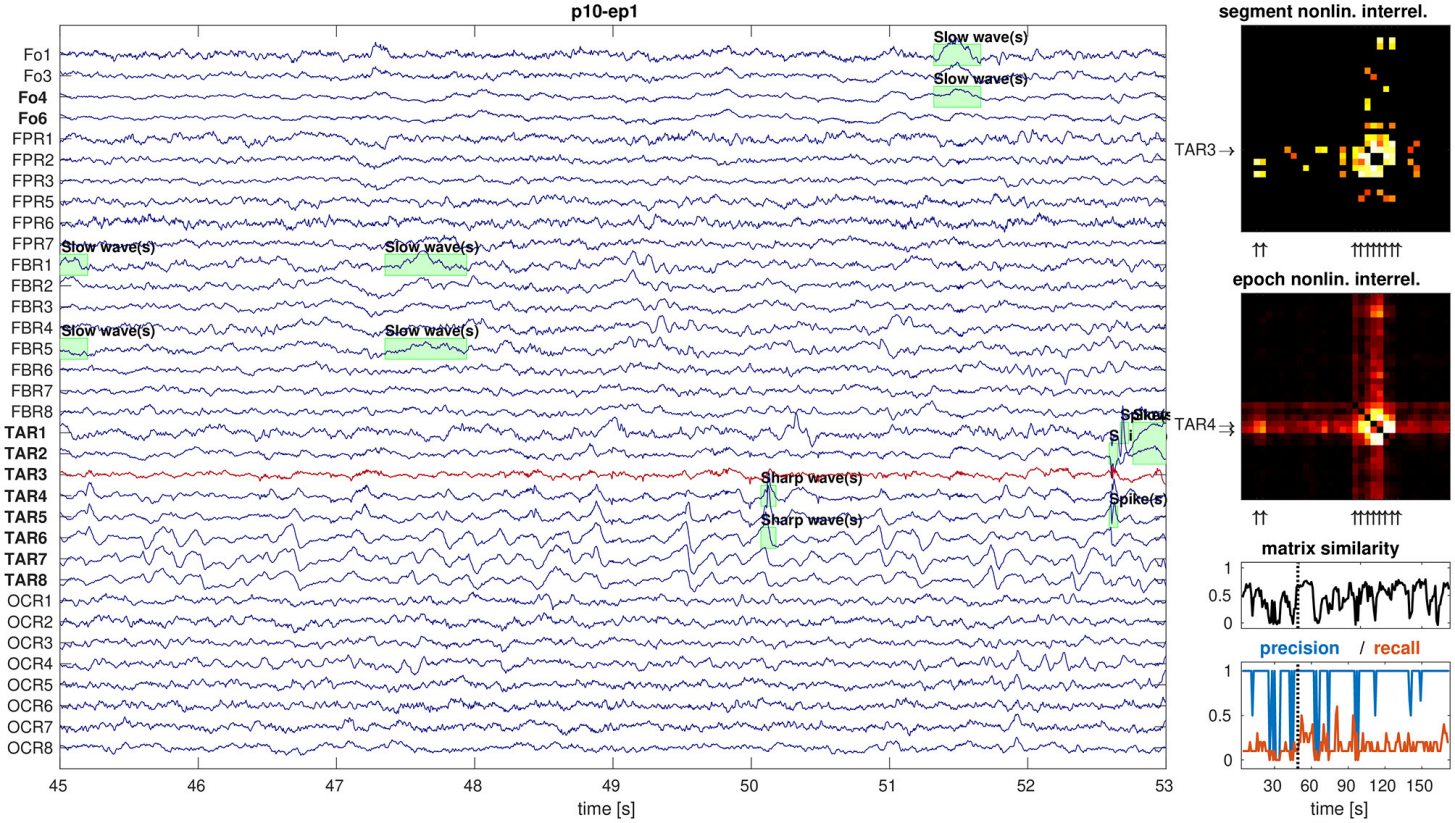
Example display of iEEG signals and corresponding non-linear excess interrelations. Shown are 8 s of preictal iEEG signals (panel 1) with various annotations of epileptiform events (green boxes). The non-linear excess interrelation matrices are shown for the selected 8-s segment (panel 2) and as average over the entire epoch of 180 s duration (panel 3). The core channels of both matrices are indicated by arrows on the respective y-axes and the selected segment's core channel TAR3 is in addition plotted in red in the EEG display. The RBT is indicated by arrows on the x-axes of the matrices and typeset in boldface in the EEG display. The similarity between all segment-wise matrices and their epoch-wise average was measured by the Pearson correlation coefficient between their elements (panel 4). High precision and low recall of the core of the selected segment to predict the RBT are representative for the entire epoch (panel 5).

In total, our data set contained 99 epochs of intracranial EEG. We found no outcome-dependent differences in the absolute numbers of artifact-free EEG channels, epoch duration, number of channels constituting the SOZ, RBT, or core channels of non-linear excess interrelation between iEEG channels (all *p*>0.09, MWU). Figure 1 illustrates the relation between iEEG waveforms, visually detectable epileptiform events (here slow and sharp waves, many of them outside the RBT) and the non-linear excess interrelations at the example of patient p10. The selected segment is representative for the total interrelation pattern and shows high precision as well as low recall regarding prediction of the RBT by channels of the core of non-linear excess interrelations or epileptiform events.

In total, 15,070 preictal epileptiform events have been included: 5,693 spikes (37.8%), 5,226 slow waves (34.7%), 3,369 sharp waves (22.4%), and 782 fast oscillations (5.2%). We found no outcome-dependent difference in average event duration, total number of channels containing events, average number of channels per event, and number of events per minute and channel (all *p* > 0.5, MWU). Likewise, we found no outcome-dependent difference in the relative portion of channels being part of the SOZ, the RBT, or the core of our qEEG analysis (all *p* > 0.2, MWU). However, there was a trend toward a higher portion of channels containing visually detectable epileptiform events in the favorable outcome group (*p* = 0.016, MWU).

**Figure 2:**
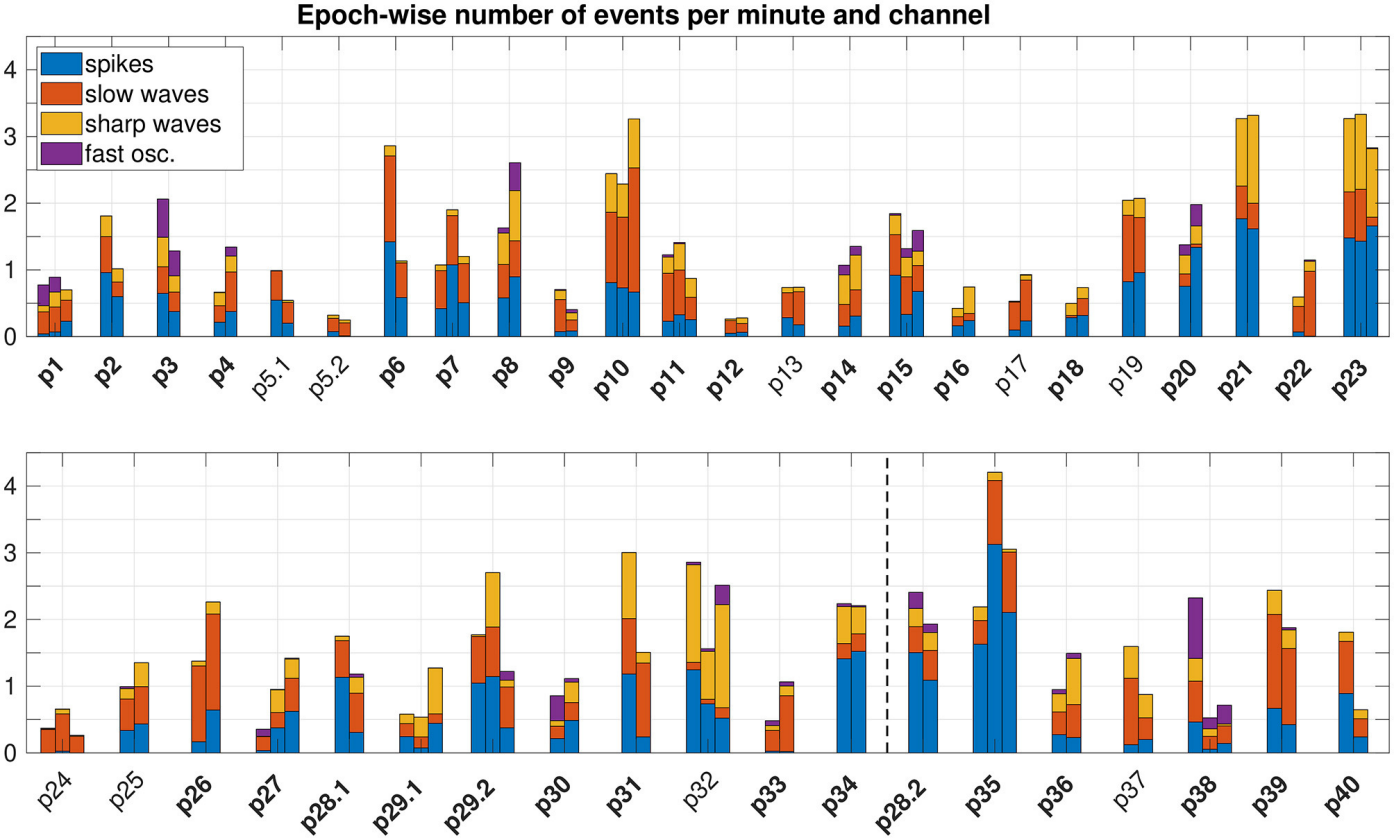
Epoch-wise frequency of preictal epileptiform events. To compare the number of events across different epoch lengths and different implantation schemes (i.e. different number of iEEG channels), we normalized to the epoch duration and total number of channels that comprised events. We did not normalize to the total number of channels implanted, because the portion of channels recording from tissue able to produce epileptiform events varied between patients. Epochs are grouped patient-wise. Patients with a favorable post-surgical outcome appear in the upper panel. The dashed vertical line in the lower panel separates patients with an unfavorable outcome (left) resp. without surgery (right). IDs of patients with temporal lobe epilepsy are plotted in bold face.

The number of epileptiform events identified before seizure onset largely varied between different patients and epochs (see Figure 2). Whereas spikes, slow waves, and sharp waves occurred in all patients, fast oscillations were present only in some. The relative partition of event types clearly differed between patients and was roughly patient-specific. In addition, a highly significant outcome-dependent difference in the relative frequencies of event-types was found (spikes, slow waves, sharp waves, fast oscillations), indicating reduced proportion of sharp waves in patients with favorable outcome (*p* = 0, Chi-Square).

After removal of patients with a non-temporal seizure onset from the main analysis (see Table 1), 44 epochs from patients with favorable outcome, 21 from patients with unfavorable outcome, and 14 from patients without surgery were used in our group-wise comparisons.

### 3.1. Do Preictal Epileptiform Events Predict SOZ, RBT or Core Channels?

**Figure 3:**
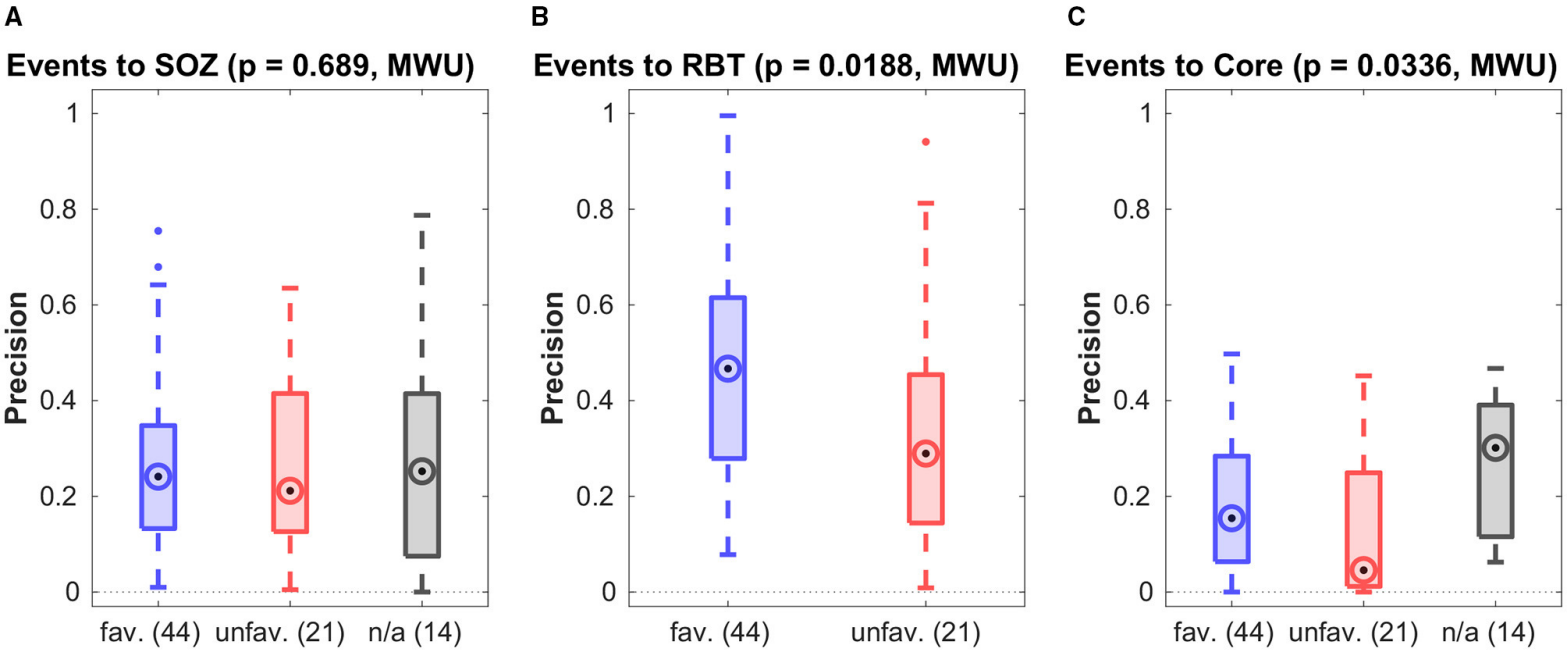
Precision of preictal epileptiform events to predict various channel sets. Results are grouped by post-surgical outcome (favorable/unfavorable) resp. those without surgery (n/a). In all panels the circled dot indicates the median of the distribution, the first (*q*1) and third quartile (*q*3) are indicated by the bottom and top edges of the box and the whiskers comprise all data points in the range *q*1−1.5*(*q*3−*q*1) to *q*3+1.5*(*q*3−*q*1). Values beyond this range are displayed as dots. The p-values for differences between the favorable and unfavorable outcome groups is indicated at the top. Similar figures for recall and F1-score can be found in the Supplementary Materials.

Figure 3 shows the precision for prediction of the SOZ, the RBT and core channels of the qEEG marker by visually detectable epileptiform events of patients with temporal lobe epilepsy regardless their type. Accuracy quantifiers are summarized in Table 2. Similar results for the small patient group with extra-temporal seizure onset are compiled in section 5 of the Supplementary Materials. General prediction power of epileptiform events for any of the channel sets was low (F1-scores below 0.37 in more than 75% of epochs). No difference was found between patients with favorable and unfavorable post-surgical seizure control for any channel set or measure (all *p* > 0.01, MWU).

**Table 2:** Distribution of epoch-wise accuracy quantifiers for predictions.

		**Favorable**	**Unfavorable**	**No surgery**	***p*** **-value**
		**median [q1, q3]**	**median [q1, q3]**	**median [q1, q3]**	**fav. vs. unfav**.
Events to SOZ	Precision	0.24 [0.13, 0.35]	0.21 [0.13, 0.41]	0.25 [0.075, 0.41]	0.689
	Recall	0.25 [0.13, 0.46]	0.19 [0.12, 0.38]	0.25 [0.1, 0.38]	0.296
	F1-score	0.24 [0.13, 0.38]	0.19 [0.12, 0.38]	0.22 [0.087, 0.41]	0.523
Events to RBT	Precision	0.47 [0.28, 0.62]	0.29 [0.14, 0.45]	n/a	0.019
	Recall	0.17 [0.098, 0.28]	0.13 [0.038, 0.18]	n/a	0.018
	F1-score	0.23 [0.15, 0.37]	0.19 [0.06, 0.27]	n/a	0.022
Events to Core	Precision	0.15 [0.064, 0.28]	0.046 [0.012, 0.25]	0.3 [0.12, 0.39]	0.034
	Recall	0.35 [0.22, 0.51]	0.13 [0.026, 0.48]	0.34 [0.25, 0.38]	0.013
	F1-score	0.24 [0.088, 0.35]	0.068 [0.016, 0.28]	0.27 [0.13, 0.35]	0.014
SOZ to RBT	Precision	1 [0.62, 1]	0.2 [0, 0.85]	n/a	<10^−3^
	Recall	0.29 [0.22, 0.45]	0.062 [0, 0.31]	n/a	0.008
	F1-score	0.44 [0.36, 0.53]	0.095 [0, 0.43]	n/a	0.004
Core to SOZ	Precision	1 [0, 1]	0 [0, 0.05]	0.5 [0, 1]	0.002
	Recall	0.25 [0, 0.5]	0 [0, 0.062]	0.29 [0, 0.8]	0.002
	F1-score	0.4 [0, 0.67]	0 [0, 0.071]	0.34 [0, 0.5]	0.001
Core to RBT	Precision	1 [1, 1]	0 [0, 0.7]	n/a	<10^−5^
	Recall	0.18 [0.077, 0.25]	0 [0, 0.11]	n/a	<10^−4^
	F1-score	0.3 [0.14, 0.4]	0 [0, 0.15]	n/a	<10^−4^

Precision was higher for the prediction of the RBT than of the SOZ (*p* = 0.0002, MWU), whereas for recall the opposite was found (*p* = 0.0022, MWU). For prediction of the RBT the precision was higher than the recall (*p* < 10^−8^, MWU), indicating that we found more FN than FP. For prediction of core channels a significant difference in the opposite direction was observed (*p* < 10^−3^, MWU) and no difference was found for prediction of the SOZ.

**Figure 4:**
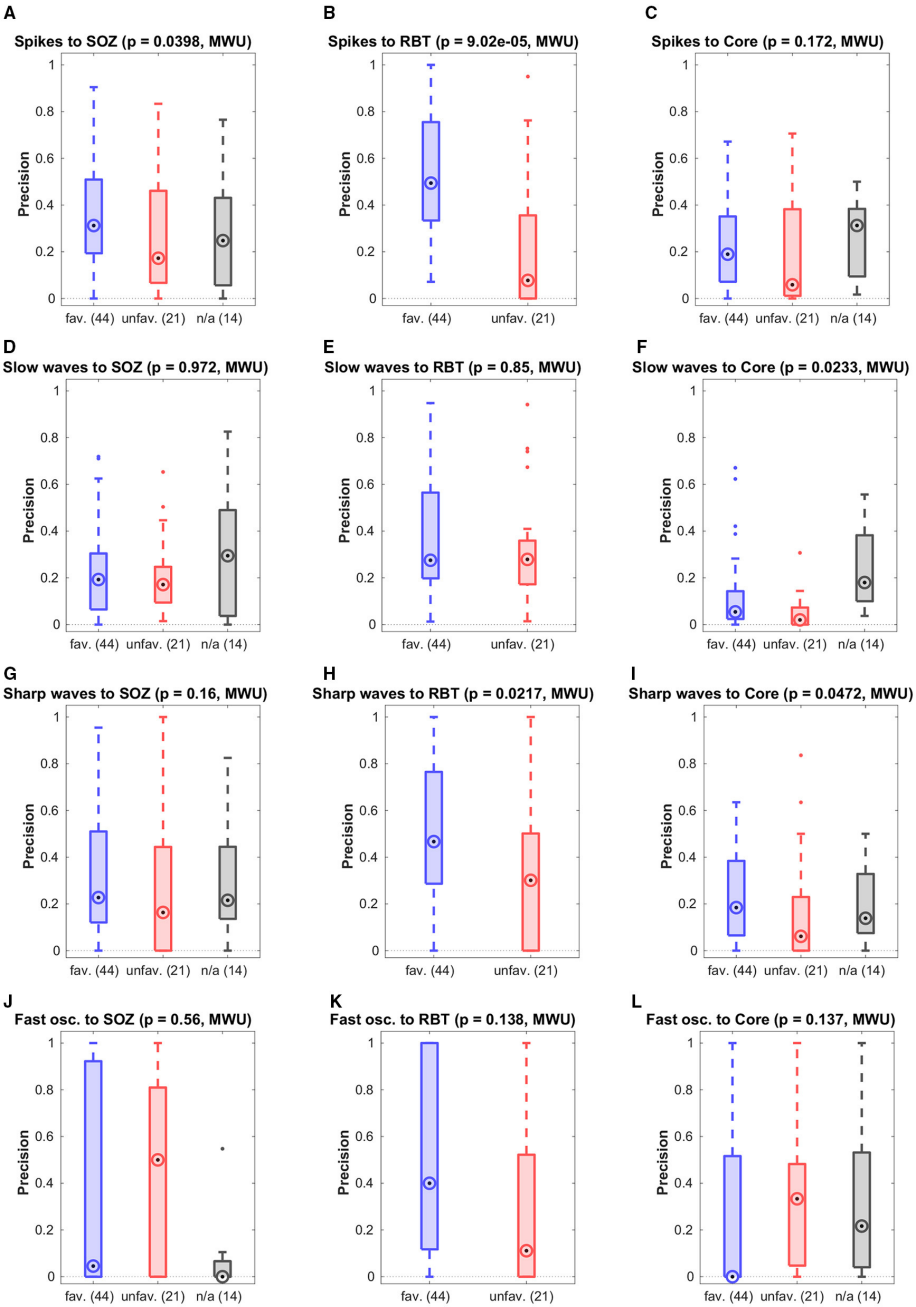
Breakdown of the prediction of channel sets by specific event types. Shown is the precision grouped by the post-surgical outcome.

Results for separate analysis of all four types of epileptiform events in patients with temporal seizure onset are presented in Figure 4. The precision for the prediction of the RBT by epileptic spikes was higher in the favorable than in the unfavorable outcome group. Apart from this exception, the observations made for event sub-types separately were not different from the pooled analysis. Specifically, none of the event subtypes was associated with the core channels of non-linear excess interrelations.

### 3.2. Can SOZ or RBT Be Predicted by Quantitative EEG Analysis?

**Figure 5:**
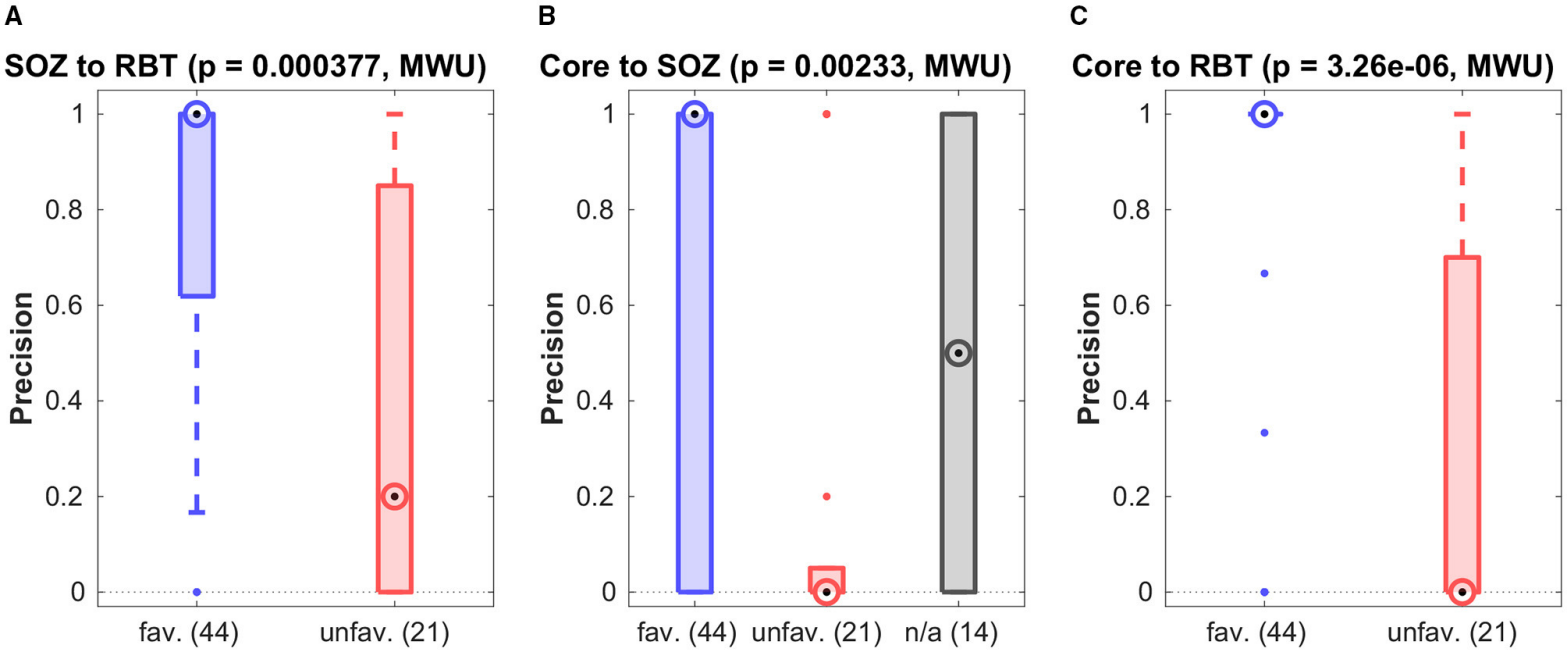
Association among various channel sets. Group-wise precision of the SOZ to predict the RBT resp. of the core to predict either of them.

For patients with temporal seizure onset, the precision for prediction of the RBT by the SOZ was high in the favorable outcome group (see Figure 5A and Table 2), and the group difference was significant (*p* = 0.0004, MWU). For recall and F1-score trends for higher values in the favorable group were observed (see Figure S6 of the Supplementary Materials for a compilation of box plots). Precision was higher than recall in the favorable outcome group (*p* < 10^−7^, MWU), again indicating that prediction of the RBT by the SOZ yielded many more FN than FP.

Figures 5B,C show precision for the prediction of the SOZ and the RBT by the core channels of the qEEG marker. In both cases the median of all accuracy measures (precision, recall and F1-score, see Figures S7, S8 of the Supplementary Materials for box plots) was zero in the unfavorable group but finite if outcome was favorable. In more than 80% of cases with favorable outcome the precision for prediction of the RBT was one, whereas in the unfavorable group it was smaller than 0.7 in 75% of cases. The group difference was significant (*p* < 10^−5^, MWU). In addition, significantly higher recall and F1-score were found in the favorable group (both *p* < 10^−4^, MWU).

For prediction of the SOZ there were outcome-dependent trends for higher precision and recall in the favorable group (*p* = 0.0023 resp. *p* = 0.0021, MWU) and a significant difference in the F1-score (*p* = 0.0014, MWU). In the favorable outcome group precision for prediction of the RBT was higher than recall (*p* < 10^−9^, MWU), again indicating that many more FN than FP were generated. No such difference was found for prediction of the SOZ. The median precision for prediction of the SOZ by core channels was also 1 in the favorable group, but the IQR was broader.

Our preliminary results in section 5 of the Supplementary Materials indicate generally lower associations among epileptiform events, the qEEG marker, the SOZ and the RBT in patients with extra-temporal seizure onset.

### 3.3. Focus on Patients With Favorable Outcome After Surgery

Reasons for unfavorable seizure control after surgery can be manifold. Since we only know with certainty that the EZ was included in the RBT if seizure freedom was reached, we analyzed the favorable outcome group in more detail. Here, the median precision of 1 for core channels of non-linear interrelation to predict the RBT was significantly higher than the value 0.47 of the epileptiform events (*p* < 10^−8^, MWU, see Figures 5B,C). Recall and F1-score were not different, though (*p* > 0.45). For prediction of the SOZ we did not find a performance difference between events and core channels (*p* > 0.025 for all accuracy quantifiers).

### 3.4. Patients With Bilateral Seizure Onset and Unilateral Resection

Two of the included patients had (unilateral) resections despite bilateral seizure onsets during presurgical evaluation. In these, no epochs preceding seizures with onset contralateral to the resection were included in the previous analyses because they occurred after the first three recorded seizures (which was a selection criterion). However, since we consider these cases as especially elucidating, we analyzed also the seizures with contralateral onset and discuss them separately.

**Figure 6:**
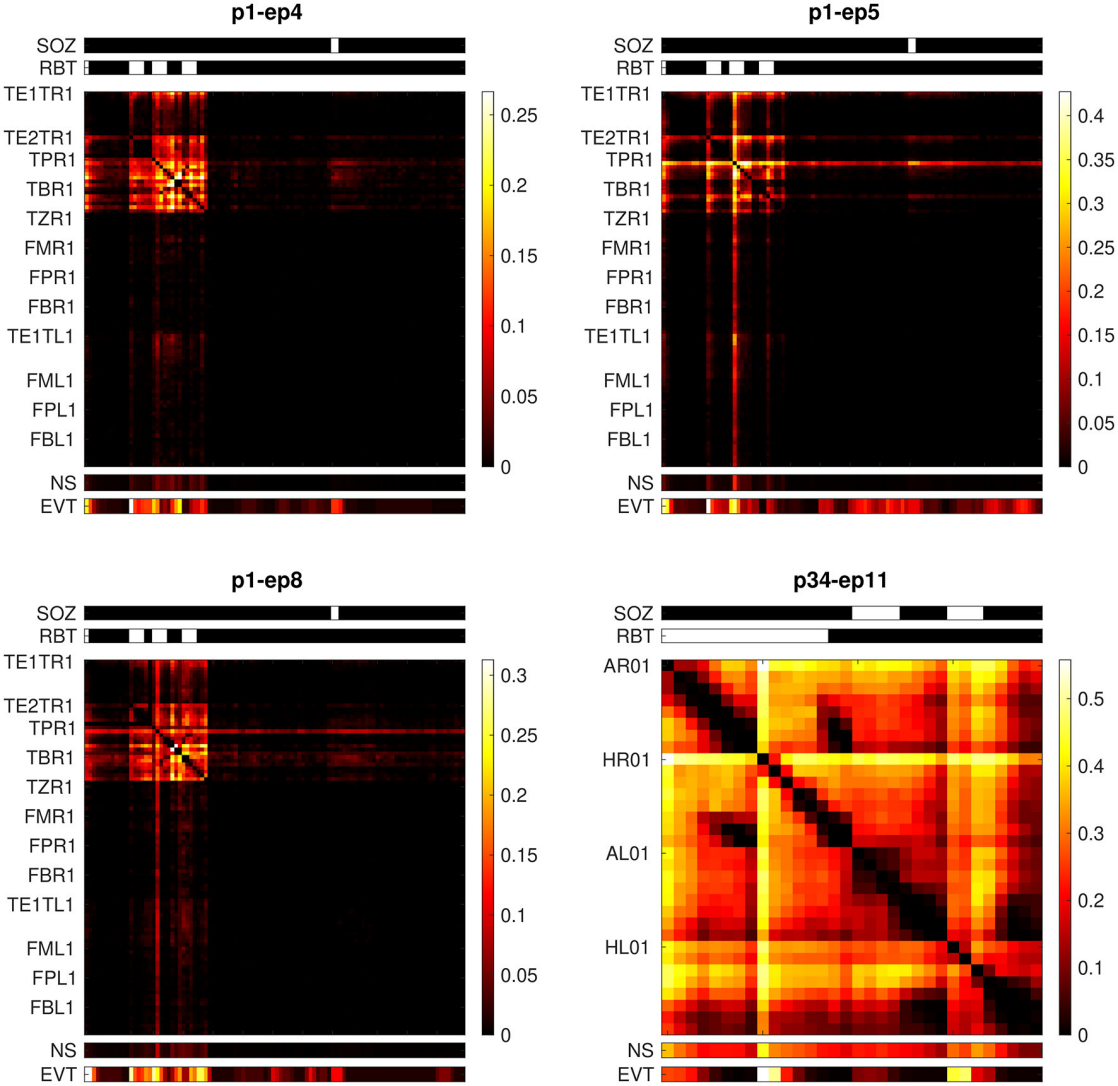
Epoch-wise averaged non-linear excess interrelation matrices of two patients with presurgical bilateral seizure onset. Shown are the epochs preceding the seizures with onset contralateral to the resection. Above the matrices, the SOZ and the RBT are indicated by white bars. Below the matrices, the node strength (NS) and the channel-wise number of events (EVT, normalized to the respective color scale) are displayed.

Patient p1 had a right-sided temporal pole-ectomy and selective amygdala-hippocampectomy and became free of disabling seizures after surgery (Engel class I). Epoch-wise averages of the non-linear excess interrelation matrices preceding the first three seizures with left-sided onset are displayed in panels 1 to 3 of Figure 6. Epileptiform events were similarly observable in the RBT and the SOZ located in different brain hemispheres. Strong non-linear excess interrelations were present in the right hemisphere and especially in the RBT but not in any of the channels recording from the left hemisphere (electrodes TE1TL, FML, FPL, and FBL). According to our hypothesis that non-linear excess interrelations could be associated with epileptogenic tissue, this suggests favorable post-surgical seizure control after a right-sided resection. This is indeed in agreement with the observed outcome.

Patient p34 was already discussed in detail in our previous study (32), see Figure 8 and associated paragraphs in section 3.4 of that publication. This patient had a right-sided selective amygdala-hippocampectomy without any subsequent worthwhile improvement (Engel class IV). In the epoch preceding the only available seizure with onset in the left hemisphere, virtually all channels recording from this hemisphere (electrodes AL and HL) show strong non-linear excess interrelations (panel 4 in Figure 6). Epileptiform events were equally dominant in the hippocampus of both hemispheres. Based on our hypothesis one would expect that these widespread non-linear excess interrelations contradict seizure freedom after surgery. Again, this matches the observed outcome.

## 4. Discussion

### 4.1. Summary

The main goal of this work was to investigate the relation between our qEEG marker non-linear excess interrelation (32, 34, 41) and preictal epileptiform events detected by a human expert in visual EEG assessment. Since the large majority of our patients had seizure onset in the temporal lobe (32/40), we restricted our analyses to these cases to increase data homogeneity and investigated patients with extra-temporal seizure onset only exploratively, see section 5 of the Supplementary Materials. We did not find a close relation between both; precision for the prediction of core channels of non-linear excess interrelation by preictal events was generally low and so were recall and F1-score (see Table 2, Figure 3C, right column of Figure 4 and Figure S5 of the Supplementary Materials). No significant separation between the favorable and unfavorable outcome groups was observed.

Our analysis revealed a significant outcome dependence of the association between the qEEG marker and the RBT (see Figure 5C) as well as an association with the SOZ (see Figure 5B and Figure S8B of the Supplementary Materials). The main effect was higher precision to predict the RBT in the favorable group (*p* < 10^−5^), confirming an observation made already in (32) based on patients with standardized mesiotemporal electrode implantations. In the present work we refined this analysis and extended to various electrode implantation schemes with depth, strip and grid electrodes placed in the temporal, frontal and parietal lobes, from which only the by far largest subgroup of patients with temporal lobe epilepsy allowed detailed analysis.

Our findings are in line with recent results of a simulation study by (45). When introducing sporadic synthetic spike-and-wave discharges into scalp EEG of healthy controls with physiologically plausible amplitudes they could not observe a relevant alteration of the network structure or strength as measured by (linear) finite-lag cross-correlation. In contrast, when comparing functional connectivity patterns between patients with infantile spasms and frequent spikes to those of healthy controls, they did find patient-specific differences. We view our own results as consistent with these findings in the sense that iEEG recorded from seizure generating brain tissue is identifyable by its altered non-linear excess interrelation (ability to predict the resection in patients with favorable outcome) but individual epileptiform events do not directly cause this interrelation pattern (no prediction of core channels by epileptiform events).

Regarding prediction of the RBT by any of (i) the epileptiform events, (ii) the SOZ, or (iii) the core channels of non-linear excess interrelation we observed a significantly smaller number of FP than FN in patients of the favorable outcome group. This implies that the predictions mainly fall inside resections that help to render patients seizure free but do not fill them entirely. This is plausible, since the RBT is known to be often larger than minimally required for surgical reasons.

For the association with the SOZ, the outcome-dependent group difference was significant for the F1-score (see Figure S7B of the Supplementary Materials) and trends were observed for precision and recall (see Figure 5B and Figure S7A of the Supplementary Materials).

Taken together, the independence of visual and qEEG markers and the better prediction of the resection in patients with favorable post-surgical seizure control provide evidence that our quantitative iEEG analysis may provide additional information about signals recorded from epileptogenic brain tissue that is not accessible to visual inspection. The more detailed examination of two patients with bilateral seizure onsets additionally support our hypotheses (see Figure 6).

The relatively weak association between SOZ and RBT (F1-score < 0.55 in more than 75% of cases, see Table 2 and Figure S6B of the Supplementary Materials) requires explanation, since the resection is usually tailored to remove the SOZ. Precision was high in the majority of cases with favorable outcome (small number of FP), whereas recall was only moderate or even small (considerable number of FN, see Table 2 and Figure 5A). This observation is consistent with the fact that despite the crucial role of the SOZ in surgery planning, the actual resection is typically more extensive for surgical reasons.

Agreement of preictal epileptiform events with the SOZ and the RBT was in general low (see Figures 3A,B, 4). Spikes were the only event sub-type that had differential predictive values in the favorable and unfavorable outcome groups, whereas neither any of the other event sub-types nor all events in conjunction did. Separation between the outcome groups was larger for prediction of the RBT than for prediction of the SOZ. This observation is remarkable, since the SOZ is determined by visual EEG assessment. However, it is crucial to note that the SOZ was defined based on the first *ictal* signal alterations, whereas the epileptiform events studied here were *preictal*. It is known that the mechanisms behind both are not necessarily identical (1, 47–49).

### 4.2. Limitations

Our study has limitations. First, our indirect argument based on contrasting the favorable (Engel classes I and II) and the unfavorable outcome groups (Engel classes III and IV) may be regarded sub-optimal, because there might be reasons for patients to experience ongoing seizures *other* than incomplete resection of the EZ (e.g., scarring or hypothetical generation of a new EZ). However, this does not affect the data points in the favorable outcome group and our main observations remain valid: The occurrence of preictal epileptiform events does in general not predict the SOZ, the RBT or the core channels of non-linear excess interrelations (see Figure 3). At the same time, the ability of core channels to predict the RBT has a median precision of 1 in the favorable outcome group (see Figure 5C), a value significantly higher than for epileptiform events.

Second, since seizure onset in our patient group was temporal in the vast majority of cases, our data did not allow to investigate a potential confounding influence of etiology. Instead, we restricted our main group analyses to temporal onset cases. Robust evaluation of patients with extra-temporal seizure onset will require collection of more such cases and remains the scope of future work.

Third, we did not explore the impact of disease duration.

Fourth, EEGs of patients in the favorable outcome group showed a trend toward a higher proportion of channels with visually detectable epileptiform events. A possible explanation is that in “easier patients” the location of the epileptogenic brain tissue was clearer a priori. Thus, also the implantation scheme and the resection were better defined. The relevance of spatial sampling for qEEG results has recently also been highlighted by (50).

Finally, we had expert annotations available only from a single rater since detailed annotating is very time consuming. Publicly available EEG data of epilepsy patients could not be used to enhance our study size for different reasons. These data are either restricted to scalp EEG [(51); isip.piconepress.com/projects/tuh_eeg/], lack at least one essential piece of information about surgery extent, outcome or detailed EEG annotations (ieeg.org, openneuro.org/datasets/ds003029/versions/1.0.2) or require payment of usage fees (epilepsy-database.eu/).

### 4.3. Conclusion

In conclusion, we herein demonstrated the potential of non-linear excess interrelations between preictal iEEG signals to provide clinically useful additional information for the planning of resective surgery in temporal lobe epilepsy. Importantly, we have shown additionally that this qEEG marker is largely independent from visually detectable preictal epileptiform events and has a higher precision for predicting the RBT than the SOZ in cases with favorable outcome. Hence, our quantitative iEEG analysis is not directly associated with established visually detectable markers of epileptogenic brain tissue, which are regarded as locally restricted phenomena. It rather captures potentially far-reaching non-linear dependencies between brain regions that seem to reflect pathological activity. This further underlines the benefit and importance to incorporate the network concept of epilepsy into presurgical patient evaluation. To foster similar research also for other qEEG makers, we made the iEEG recordings and human annotations used in this paper publicly available. Furthermore, we encourage the scientific community to provide independent expert annotations of these recordings using our custom EEG reader to enable comparison also with inter-rater agreement.

## Data Availability Statement

The EEG reader and the raw data supporting the conclusions of this article are available via GitHub: https://github.com/SCAN-NRAD/scanEEGviewer.

## Ethics Statement

The studies involving human participants were reviewed and approved by Ethics Committee of the Canton of Bern. Written informed consent to participate in this study was provided by the participants' legal guardian/next of kin.

## Author Contributions

CR: study design. MD: data annotation. MM and CR: data analysis and writing of manuscript. KS and CR: data curation. MM: writing of software. MM, MD, KS, RW, and CR: final review of manuscript. All authors contributed to the article and approved the submitted version.

## Funding

This study was supported by the Swiss National Science Foundation (SNSF) (grant no: CRSK-3_190817/1).

## Conflict of Interest

The authors declare that the research was conducted in the absence of any commercial or financial relationships that could be construed as a potential conflict of interest.

## Publisher's Note

All claims expressed in this article are solely those of the authors and do not necessarily represent those of their affiliated organizations, or those of the publisher, the editors and the reviewers. Any product that may be evaluated in this article, or claim that may be made by its manufacturer, is not guaranteed or endorsed by the publisher.
