# Supplementary material for: More Than Spikes: On the Added Value of Non-linear Intracranial EEG Analysis for Surgery Planning in Temporal Lobe Epilepsy
Source: Front Neurol. 2022 Jan 13;12:741450. doi: 10.3389/fneur.2021.741450 (PMC8793863; doi:10.3389/fneur.2021.741450)
Supplement: Supplementary file 1 [file Presentation_1.pdf]

More than spikes: on the added value of nonlinear iEEG analysis for surgery planning in temporal lobe epilepsy

Supplementary Material

Michael Müller[1], Martijn Dekkers[2], Roland Wiest[1], Kaspar Schindler[2], and Christian Rummel *[1]

[1]Support Center for Advanced Neuroimaging (SCAN), University Institute for Diagnostic and Interventional Neuroradiology, Inselspital, Bern, Switzerland
[2]Department of Neurology, Inselspital, Bern University Hospital, University Bern, Bern, Switzerland

*Corresponding author: Support Center for Advanced Neuroimaging (SCAN), University Institute for Diagnostic and Interventional Neuroradiology, Inselspital, Freiburgstrasse 4, 3010 Bern, Switzerland, Tel.: +41 (0)31 632 80 38, Email: crummel@web.de

1 Processing pipeline

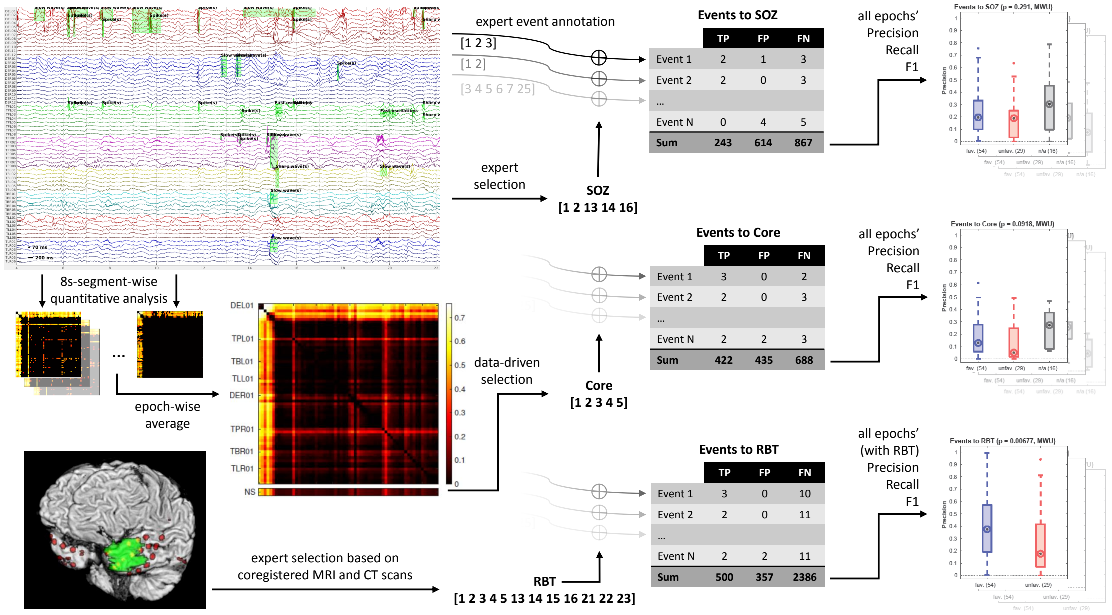

Figure S1: Visualization of the processing pipeline.
Shown is the association of preictal epileptiform events to the SOZ, the RBT, and the core channels of nonlinear excess interrelations. Numbers in brackets refer to specific sets of iEEG-channels. Congruence resp. discrepancy between sets was pooled over all events of an epoch and sums were used to calculate precision, recall, and F1-score of all epochs, which were then grouped based on the post-surgical outcome for comparisons. Associations among SOZ, RBT, and the core were assessed analogously without any pooling.

2 Accuracy quantifiers

Definitions of the herein used accuracy quantifiers precision (Equation 1), recall (Equation 2), and their harmonic mean the F1-score (Equation 3).

$$Precision = \frac{TP}{TP + FP} \tag{1}$$

$$Recall = \frac{TP}{TP + FN} \tag{2}$$

$$F_1 = \frac{2 * Precision * Recall}{Precision + Recall} = \frac{TP}{TP + \frac{FP+FN}{2}} \tag{3}$$

3 EEG to Core Example

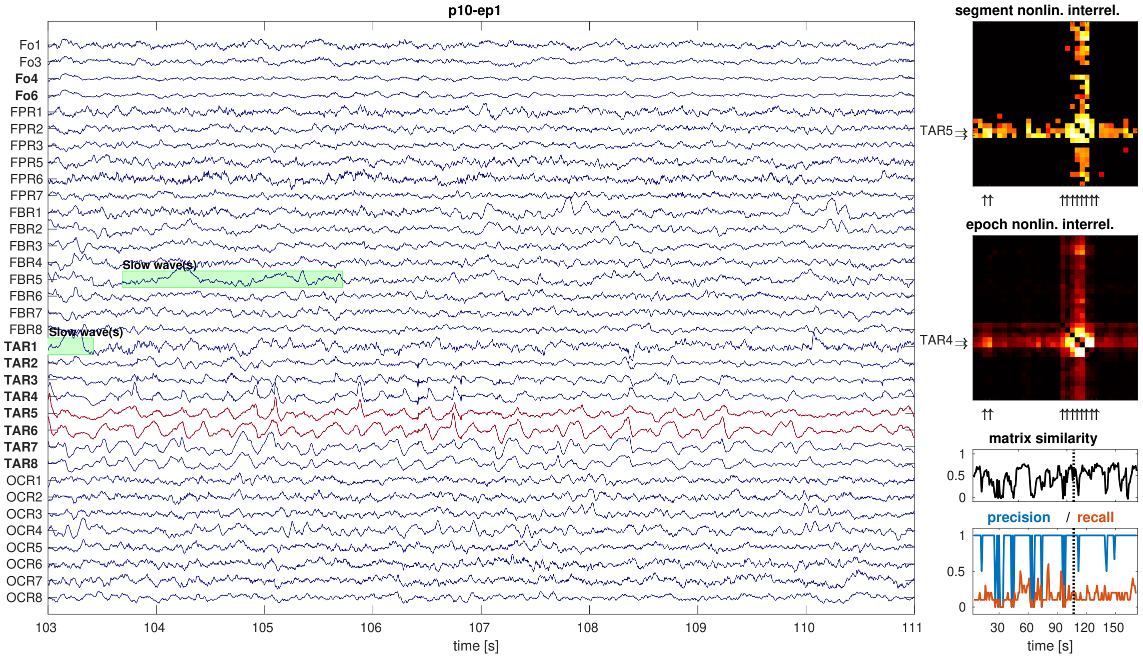

Figure S2: Additional example similar to Figure 1 of the main text for another time point. Although almost no visually detectable epileptiform events are present in this segment, the core structure is still very similar to the one of the entire epoch and the one of the segment shown in the main text (Figure 1).

4 Patients with temporal lobe epilepsy

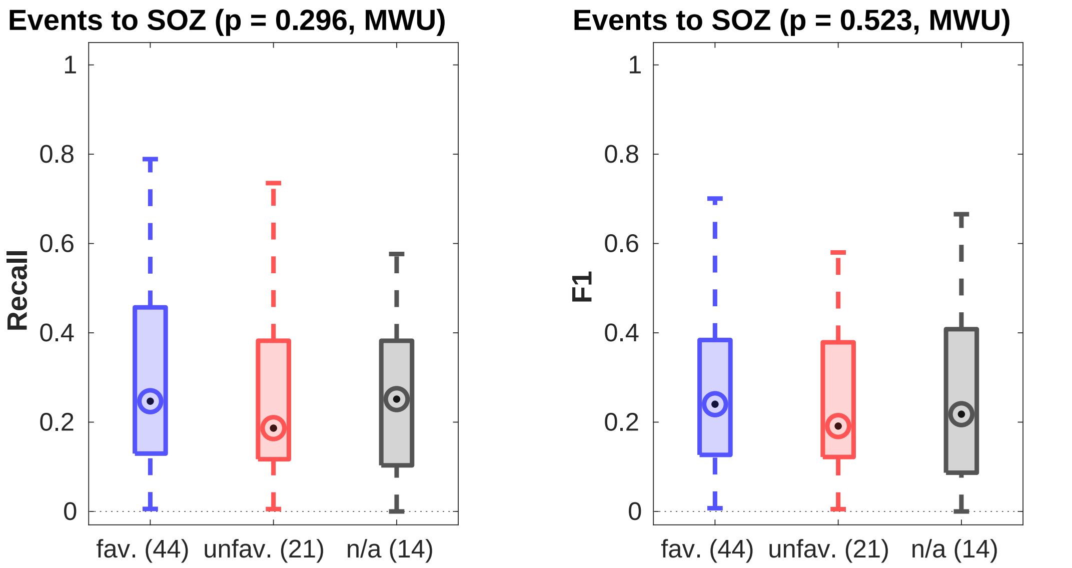

(A) (B)

Figure S3: Outcome-dependent group wise analyses of recall and F1-score indicating how much event occurences indicate the SOZ.

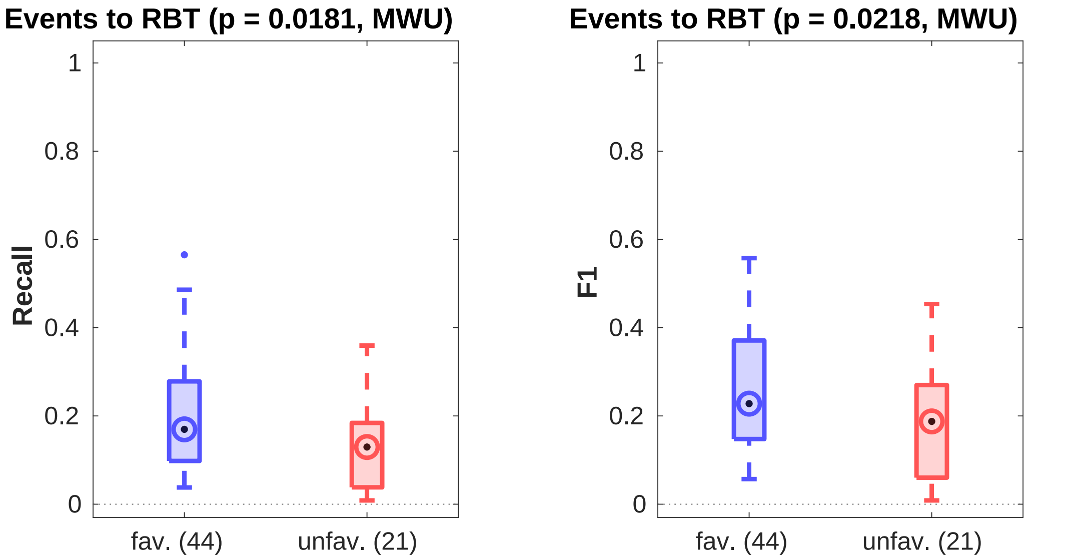

(A) (B)

Figure S4: Outcome-dependent group wise analyses of recall and F1-score indicating how much event occurences indicate the resection.

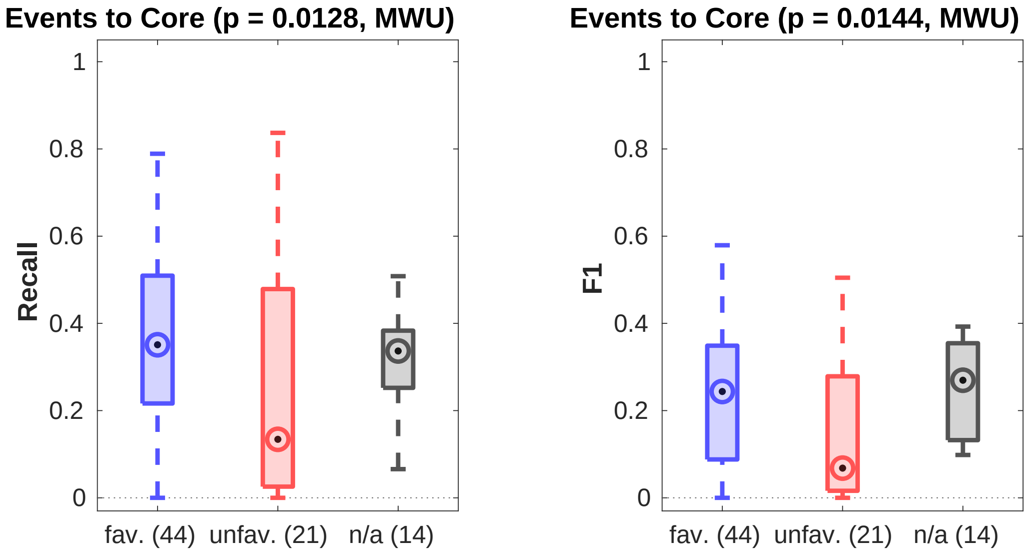

(A) (B)

Figure S5: Outcome-dependent group wise analyses of recall and F1-score indicating how much event occurences indicate the core channels.

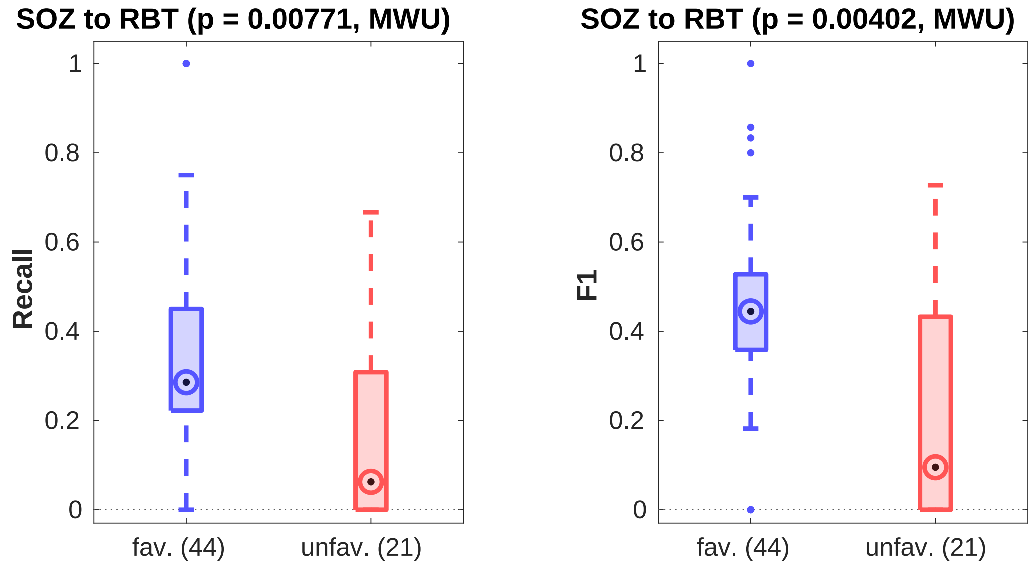

(A) (B)

Figure S6: Outcome-dependent group wise analyses of measures quantifying the relation between SOZ and resection.

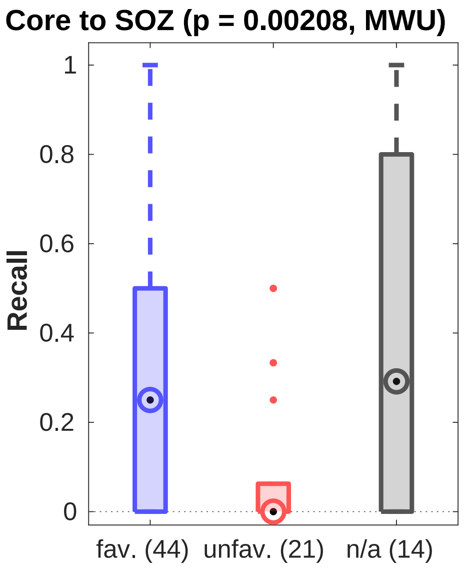

(A)

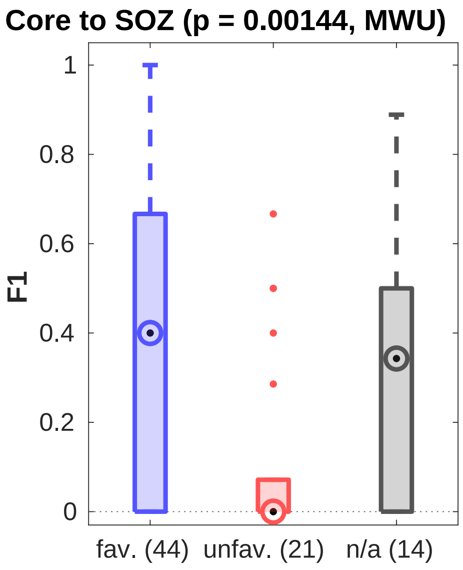

(B)

Figure S7: Outcome-dependent group wise analyses of measures quantifying the relation between core and SOZ.

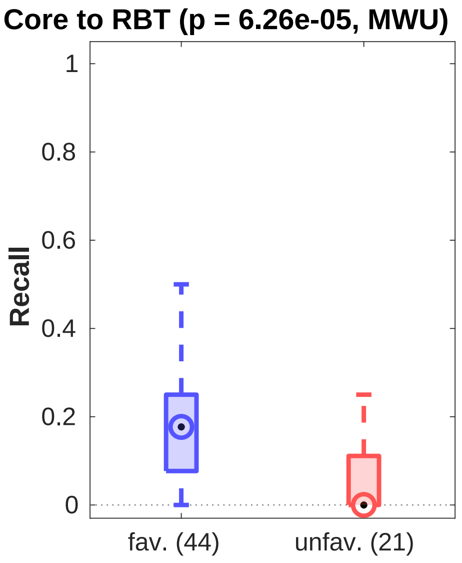

(A)

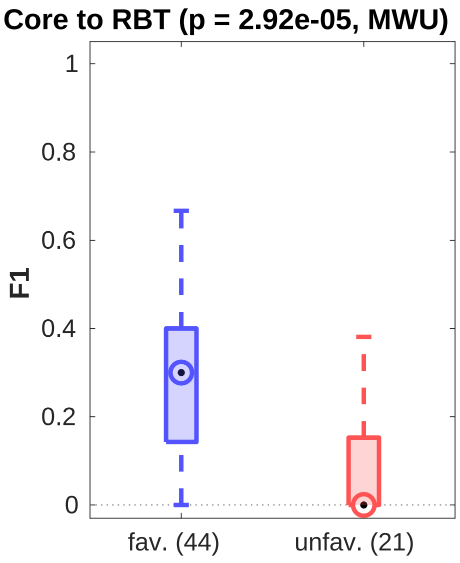

(B)

Figure S8: Outcome-dependent group wise analyses of measures quantifying the relation between core and resection.

5 Patients with extra-temporal lobe epilepsy

Group analyses for patients with seizure onset in the frontal ($N = 6$) and the parietal lobe ($N = 2$, see Table 1). To enable direct comparison with the representation for the larger group of patients with temporal lobe epilepsy ($N = 32$), we use boxplots also here despite the small sample number.

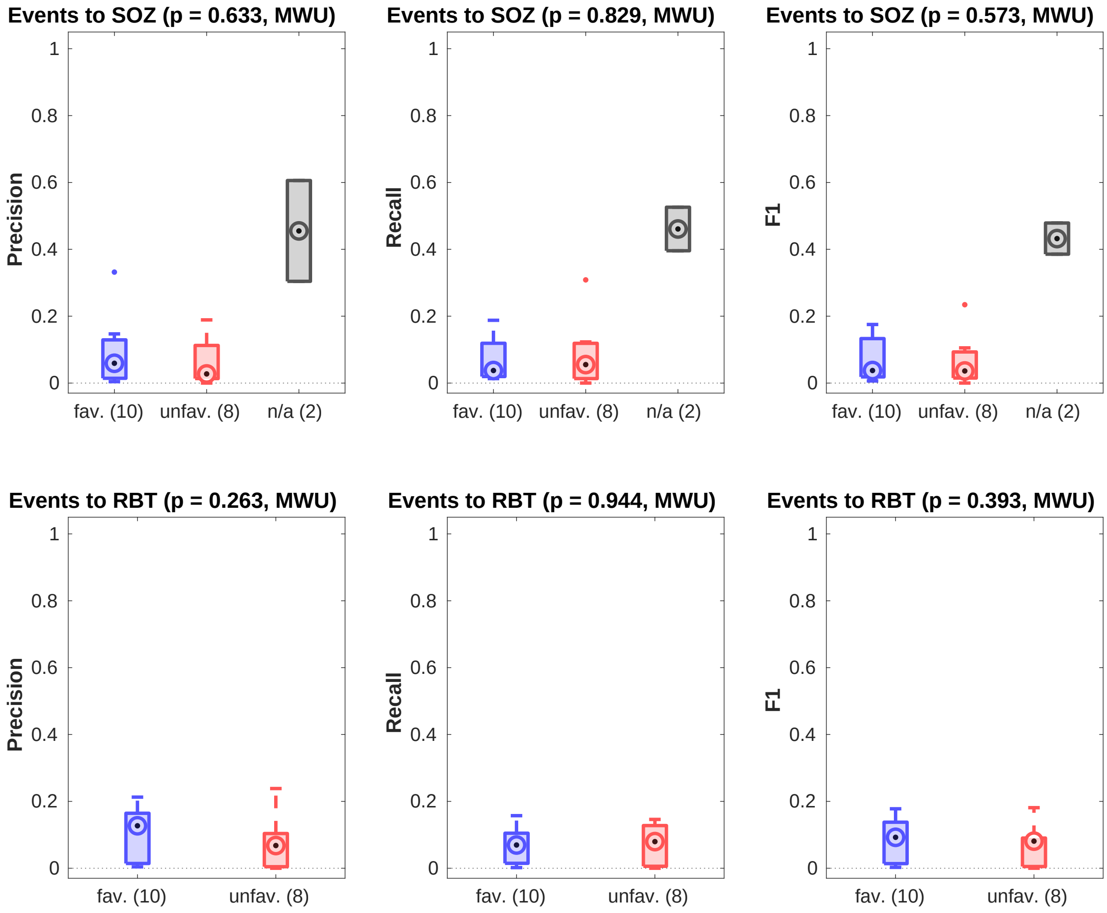

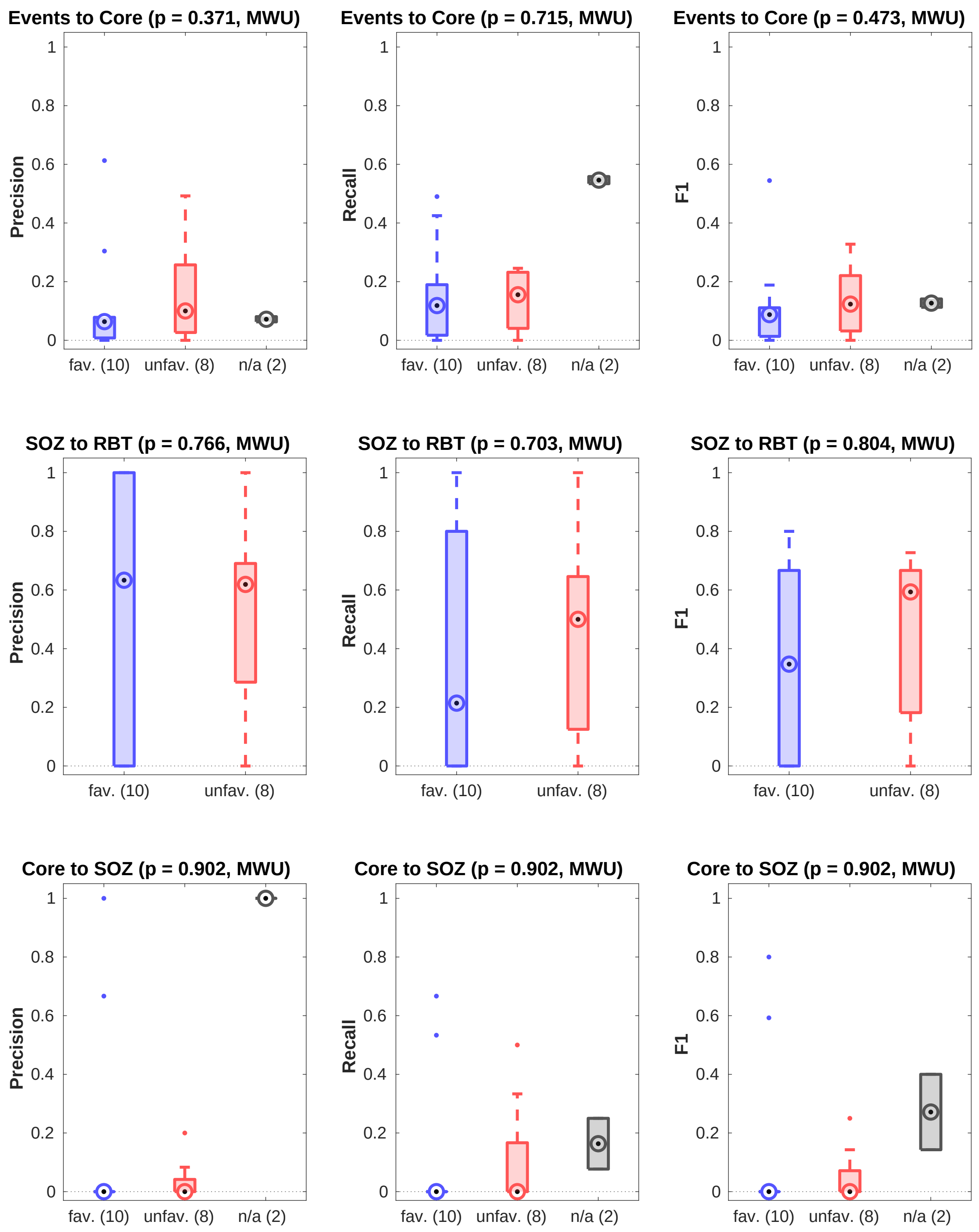
Events to Core (p = 0.371, MWU)
Events to Core (p = 0.715, MWU)
Events to Core (p = 0.473, MWU)
SOZ to RBT (p = 0.766, MWU)
SOZ to RBT (p = 0.703, MWU)
SOZ to RBT (p = 0.804, MWU)
Core to SOZ (p = 0.902, MWU)
Core to SOZ (p = 0.902, MWU)
Core to SOZ (p = 0.902, MWU)
Precision
Recall
F1
fav. (10)
unfav. (8)
n/a (2)
0
0.2
0.4
0.6
0.8
1

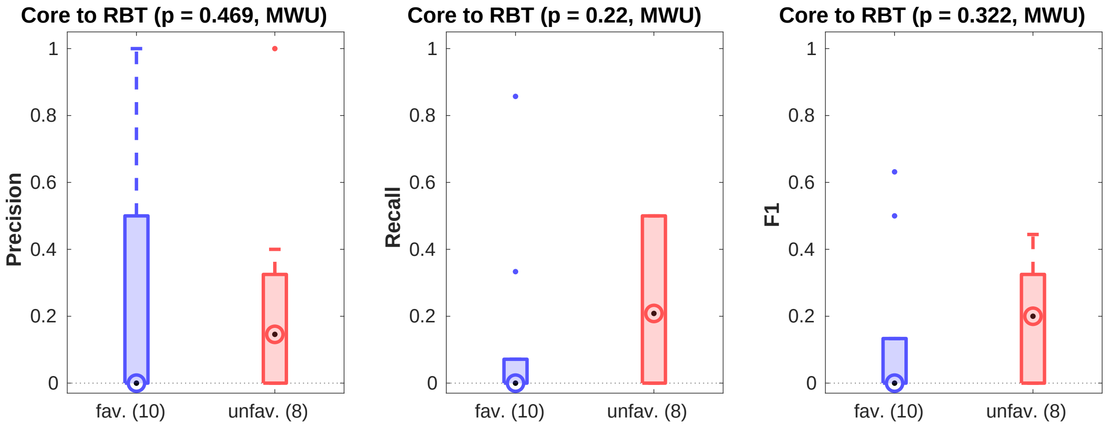
Core to RBT (p = 0.469, MWU)
Precision
fav. (10)
unfav. (8)
Core to RBT (p = 0.22, MWU)
Recall
fav. (10)
unfav. (8)
Core to RBT (p = 0.322, MWU)
F1
fav. (10)
unfav. (8)
